# Maternal Knowledge, Attitudes, and Practices Concerning Interpregnancy Interval

**Published:** 2018-11-29

**Authors:** Carolyn R. Ahlers-Schmidt, Nikki Keene Woods, Danielle Bradshaw, Anna Rempel, Matt Engel, Mary Benton

**Affiliations:** 1 University of Kansas School of Medicine-Wichita, Department of Pediatrics, Wichita, KS; 2 Wichita State University, Department of Public Health Sciences, Wichita, KS

**Keywords:** maternal-child health services, pregnancy, reproductive history, birth intervals, infant mortality

## Abstract

**Introduction:**

Few studies have examined maternal intentions and practices related to interpregnancy interval (IPI). IPI less than 18 months has been linked to increased preterm birth and infant mortality. This manuscript reports on a cross-sectional survey of mothers conducted to understand maternal knowledge, attitudes, and practice of IPI in Sedgwick County, Kansas.

**Methods:**

New and expectant mothers and mothers of neonatal infant care unit (NICU) graduates (n = 125) were surveyed regarding the issues surrounding IPI. Front desk staff handed out self-administered surveys, which were returned to a nurse upon completion. NICU participants were emailed a link to the survey hosted on SurveyMonkey^®^.

**Results:**

Fewer than 30% of mothers reported previously receiving information about IPI from any source. When asked about risks associated with IPI, women frequently (n = 58, 45%) identified increased risk for birth outcomes with no known association with short IPI. Findings regarding maternal attitudes surrounding optimal IPI were mixed with many mothers defining ideal IPI as less than 18 months (n = 52, 42%), while broadly reporting they believed that a woman’s body needs time to heal between pregnancies. Respondents from the NICU sample generally reported shorter optimal IPI values than the other participants. When IPI was estimated from participants’ past pregnancies, half of IPIs were less than 18 months. Mothers reported they favored healthcare providers as a source for IPI education. Face-to-face discussions or printed materials were the preferred modes of education.

**Conclusions:**

Women were aware of the need for spacing between pregnancies, however, that knowledge was unassociated with past behavior. These findings should be taken into consideration when formulating future interventions.

## INTRODUCTION

The infant mortality rate in the United States (U.S.) historically has been higher than other developed countries.^1^ Reducing this rate requires innovative multifaceted interventions and interdisciplinary collaborations. Interpregnancy interval (IPI) less than 18 months is associated with increased odds of infant mortality^2^,^3^ and other adverse birth outcomes, including preterm birth and low birth weight.^3^–^7^

In Sedgwick County, Kansas, the local infant mortality rate (7.2 per 1,000 live births) has been consistently higher than Kansas and U.S. rates.^8^ Short IPI was identified as a risk factor, especially for African Americans, using perinatal periods of risk (PPOR) analysis.^9^ This analytic framework uses vital statistics (Sedgwick County 2008 – 2012) to elucidate causes of infant mortality by separating deaths into categories based on birth weight and age at death.^10^ Based on these analyses, nearly 150 fetal infant deaths may have been avoidable, including a disparity with regard to race. Both non-Hispanic Black and Hispanic infants were at increased risk of death compared to White non-Hispanics. These findings, combined with birth outcome data from these populations^11^ suggested that one of the local contributors to stillbirth and sudden and unexpected infant death was inadequate IPI.

Few studies have examined maternal intentions and practices related to IPI. A qualitative study of a diverse group of low-income postpartum women found knowledge gaps regarding outcomes associated with short IPI.^12^ Similar findings were reported in a study that interviewed mothers of premature infants in the neonatal intensive care unit (NICU).^13^

This manuscript reports on a cross-sectional survey of mothers conducted to understand maternal knowledge, attitudes, and practice of IPI in Sedgwick County.

## METHODS

### Participants

Participants included a convenience sample of mothers of infants less than one year of age and pregnant women (hereafter mothers) attending a combined obstetrical/pediatric primary care resident/faculty clinic between September and December 2015. The clinic was selected as it serves many minority patients and only 11% of the county identified as African American.^11^ Clinic participants were eligible if they had a scheduled appointment for themselves or for their infant. Front desk staff handed out self-administered surveys, which were returned to a nurse upon completion.

Mothers of NICU graduates who delivered in 2015 also were enrolled. The 85-bed, level III NICU is located in the hospital that provides 80% of the deliveries in Sedgwick County. The NICU was selected to target those with a likelihood of recent preterm delivery, a complication of short IPI. NICU participants were emailed a link to the survey hosted on SurveyMonkey^®^ (SurveyMonkey Inc., Palo Alto, CA).

Mothers who were less than 18 years old, unable to understand written English, or unable to consent were excluded. The study was approved by institutional review boards at the University of Kansas School of Medicine-Wichita, Wichita State University, and the Wichita Medical Research and Education Foundation.

### Measurement

Because no existing tool was identified, a 23-item Interpregnancy Interval Knowledge, Attitudes, and Practice survey was developed. Items were based on a review of the literature and input from medical professionals and qualitative researchers. The survey was assessed by an expert panel for wording, complexity, and content validity.

Knowledge questions (n = 3) addressed perception of recommended IPI and its associated impact on pregnancy outcomes. Attitude questions (n = 7) included preferred IPI, contributing factors for this choice and perceived influence of recommendations from healthcare providers. Practice (n = 6) was assessed using actual IPI history. Since mothers were anticipated to not know their conception date, IPI was estimated from birthdates of previous children, allowing conservatively for 28 weeks gestation if mother’s reported that their infant was born premature or 37 weeks gestation otherwise. Demographic questions (n = 7) included age, race/ethnicity, marital status, income, and insurance type.

### Analysis

Analyses were conducted in SPSS (SPSS Version 22, Armonk, NY: IBM Corp.).

## RESULTS

### Demographics

Participants included 147 mothers (Table 1). Of these, 22 were excluded for missing key questions or more than 50% of responses. Women had given birth to 0 to 7 children (median = 2); 13 women (11%) were pregnant at the time of the survey. Parity ranged from 0 to 15 (median = 2). Mothers were white (61%) with a high school education or less (43%). Mothers from the NICU were significantly older (t(120) = 4.2, p < 0.001) and differed in race/ethnicity (χ2(4, N = 123) = 21.8, p < 0.001), marital status (χ2(3, N = 123) = 22.1, p < 0.001), and insurance (χ2(5, N = 122) = 50.4, p < 0.001).

### Knowledge

Mothers were queried about their knowledge regarding a set of complications that were associated with short IPI. No mother accurately categorized all complications related to short IPI, and the average mother was unable to categorize correctly half of the complications. The list of complications had only four items related to short IPI (small for gestational age, congenital malformations, difficult child birth, and preterm labor); however, 58 mothers (45%) attributed at least one unrelated complication to short IPI (e.g., morning sickness, postpartum depression).

In total, 34 mothers (27%) reported receiving prior information on IPI. Mothers from the NICU were no more likely than those from the primary care clinic to have received information (χ2(1, N = 125) = 3.8, p = 0.052). Those who received information most often identified medical providers as their source. Family, friends, and media were reported less frequently as sources of this information (Table 2).

### Attitudes

Many mothers (43%) believed an appropriate IPI to be less than 18 months, with an additional 5% believing ideal IPI should be greater than 60 months (Table 3). NICU mothers reported generally shorter optimal perceived IPI, with 56% advocating for IPI less than 18 months (Exact, p = 0.005); however, no difference was found when the question was phrased in terms of how far apart in age they would like their children (Exact, p = 0.663). For the latter category, 99 mothers (79%) reported that they would desire an age gap of at least two years between their children.

The most frequently cited reason for perceived optimal IPI length was to allow for physical healing between pregnancies (53%), followed by time to nurture current infant (21%), personal preferences (16%), and sibling interactions (3%). However, when the question was framed in terms of differences in children’s ages, the reasoning differed, with 29% of mothers identifying sibling interactions, followed by personal preference (21%), nurturing current infant (19%), and maternal health (10%). Of reasons provided by respondents, only desire for siblings to be close in age was associated significantly with perceived optimal IPI. In all four cases, mothers reported perceived optimal IPI as less than 18 months (Exact, p = 0.028). Mothers who wanted children close in age were nearly three times as likely to endorse delivering their children less than 24 months apart (χ2(1, N = 125)=10.0, p = 0.002).

### Practice

Of the 62 mothers reporting more than one child (excluding multiples), 60 provided their children’s birthdates. Estimating IPI from these birthdates, 30 (50%) reported short IPI for at least one pregnancy. Additionally, 11 (18%) had at least one pregnancy preceded by an IPI greater than 60 months. Reported history of short IPI was unassociated with perceived optimal IPI less than 18 months (χ2(1, N = 60) = 0.7, p = 0.417).

Data on beliefs and histories did not support associations between race/ethnicity and endorsement of short IPI (χ2(4, N = 123) = 0.2, p = 0.237). Further, only 33% of African Americans in our sample reported a short IPI, not significantly different from our total sample (Exact, p = 0.472). Similarly, 23% of African American respondents reported appropriate IPI as less than 18 months (χ2(1, N = 123) = 0.2, p = 0.235).

### Education planning

Mothers preferred learning about IPI from their healthcare providers, including pediatricians (Table 4). They were less inclined to want to receive information from television, books/magazines, or the internet. Most mothers favored learning about IPI shortly after baby’s birth or before any pregnancy. Mothers desired to hear about IPI in a face-to-face discussion, or from a brochure or handout, rather than other media sources (video, email, social media, or text message).

## DISCUSSION

Sedgwick County mothers had varied knowledge, attitudes, and practice related to IPI. As demonstrated by previous literature, knowledge about possible negative health consequences associated with a short IPI often was lacking.^12^ One study found, while nearly 70% of mothers with infants in the NICU had heard of risk factors for preterm birth, such as smoking and infection, less than 32% had heard of short IPI.^13^

In terms of attitudes, most mothers recognized the importance of allowing their body to heal between pregnancies, but underestimated the time needed to do so. However, when the question was phrased in terms of differences in child age, maternal health was less likely to be reported as a decision factor, suggesting pregnancy interval may be a better way to frame educational statements related to birth spacing. Further, mothers who expressed interest in having children close in age were significantly more likely to endorse a short IPI and may be a priority group for future education.

Birth spacing was inconsistent among women, with half experiencing a short IPI and nearly 20% waiting more than five years between pregnancies. About one third of U.S. pregnancies experience short IPI, suggesting our sample may represent a population with greater propensity toward IPI of less than 18 months.^14^ Study findings were not consistent with literature^4^ or PPOR findings^10^ reporting an association between African American race and short IPI.

The majority of respondents wanted to hear about birth spacing shortly after the baby’s birth. While for many mothers, the six-week post-partum visit is sufficient for discussion and provision of birth control, for others it may be too late to prevent subsequent high-risk pregnancy. In interviews with general practitioners in the United Kingdom, doctors expressed concern that, for lower socioeconomic status and younger women, sexual activity has resumed by six weeks.^15^ In addition, many of these women miss their six-week appointment.

Such results have led to considerations of alternative solutions, such as a family planning clinic adjacent to the NICU^13^, insertion of long-acting reversible contraceptives at delivery, or co-occurrence of mother’s contraceptive care with the well-baby visit.^16^ Mothers from the current study reported they would trust their healthcare providers, including pediatricians, to share information with them regarding birth spacing during face-to-face interactions. All providers who deliver health services to new mothers should promote healthy IPI. Pediatricians may be an untapped resource for providing accurate IPI information and family planning conversations, as infants attend a greater number of appointments, including several before six weeks of age. Interventions designed to leverage this trusted relationship to provide IPI information should be considered. Of note, one of the NICU participants in this study reported learning about IPI from the hospital. Similar to prior interventions to promote safe sleep^17^,^18^ and breastfeeding^19^ in the NICU setting, it may be prudent to engage the neonatal providers in promoting optimal IPI.

There are potential barriers to adoption of appropriate IPI. This study observed that mother’s perception of optimal IPI was not associated significantly with their reported practices. While this study did not access the medical records of included mothers to confirm their reported IPI, these data suggested mothers may get pregnant sooner than they intended. This is congruent with previous literature suggesting that nearly 50% of pregnancies in the U.S. are unplanned.^20^ The authors suggest that health systems should provide education for all mothers in the perinatal period at multiple touch points, and provide resources, including comprehensive contraception counseling, to help mothers maintain a healthy IPI.

### Limitations

This study is limited as it presents a convenience sample of mothers from a combined obstetrical/pediatric clinic and a single NICU. The NICU sample was less ethnically diverse and higher educated than clinic mothers, which may be due to sampling bias related to electronic survey methods. The self-reported nature of the data and the fact that IPI was estimated from reported birthdates also were limitations.

## CONCLUSION

Maternal knowledge of IPI is less than optimal and may be improved through direct communication with healthcare providers, especially pediatricians. However, knowledge and intentions did not correspond necessarily with practice, suggesting additional barriers may exist for women looking to adhere to birth spacing recommendations. Increased birth spacing interventions may address short IPIs and improve the health of infants and mothers.

## Figures and Tables

**Table 1:** Participant characteristics.

Characteristic	Clinic (n = 83)	NICU (n = 42)	All Participants (n = 125)
Age*, mean (SD)	25.8 (5.5)	30.1 (4.9)	27.2 (5.7)
Race/Ethnicity*, n (%)			
White	39 (48)	36 (88)	75 (61)
Hispanic	24 (29)	2 (5)	26 (21)
Black	12 (15)	1 (2)	13 (11)
Mixed Race	5 (6)	0 (0)	5 (4)
Other (please specify)	2 (2)	2 (5)	4 (3)
Education, n (%)			
Less than High School Diploma	16 (19)	2 (5)	18 (14)
GED/High School Diploma	34 (41)	2 (5)	36 (29)
Some College	24 (29)	11 (26)	35 (28)
Associate’s Degree	2 (2)	6 (14)	8 (6)
Bachelor’s Degree	4 (5)	15 (36)	19 (15)
Master’s Degree or greater	2 (2)	6 (14)	8 (6)
Other	1 (1)	0 (0)	1 (1)
Marital Status*			
Married	29 (36)	33 (79)	62 (50)
Not married, but in a relationship	29 (36)	6 (14)	35 (28)
Never married	19 (23)	1 (2)	20 (16)
Divorced/separated	4 (5)	2 (5)	6 (5)
Insurance Coverage*			
Covered by Medicaid	62 (78)	7 (17)	69 (57)
Insurance provided through baby’s father’s employer	8 (10)	17 (40)	25 (20)
Insurance provided through baby’s mother’s employer	5 (6)	14 (33)	19 (16)
Other private insurance	1 (1)	3 (7)	4 (3)
Don’t know	4 (5)	0 (0)	4 (3)
Other	0 (0)	1 (2)	1 (1)

* Significantly different between groups; p < 0.05.

**Table 2:** Source of IPI information among mothers who previously had received information on IPI.

	Clinic (n = 18)	NICU (n = 16)	All Participants (n = 34)
Health care providers			
Obstetrician (OB)	9 (47)	6 (38)	15 (43)
Family doctor	6 (32)	6 (38)	12 (34)
Hospital	4 (21)	1 (6)	5 (14)
Pediatrician	2 (11)	1 (6)	3 (9)
Nurse	1 (5)	1 (6)	2 (6)
Midwife	0 (0)	0 (0)	0 (0)
Doula	0 (0)	0 (0)	0 (0)
Other healthcare worker	0 (0)	2 (13)	2 (6)
Friends/Family			
Family	7 (37)	4 (25)	11 (31)
Friends	3 (16)	4 (25)	7 (20)
Media			
Internet	4 (21)	3 (19)	7 (20)
Books/magazines	2 (11)	4 (25)	6 (17)
Television	1 (5)	1 (6)	2 (6)
Other	1 (5)	3 (19)	4 (11)

**Table 3:** Mother’s beliefs/desires about IPI.

	Clinic (n = 18)	NICU (n = 16)	All Participants (n = 34)
Perceived ideal IPI*			
Less than 6 months	0 (0)	1 (2)	1 (1)
6 – 11 months	7 (8)	2 (5)	9 (7)
12 – 17 months	23 (28)	19 (45)	42 (34)
18 – 23 months	14 (17)	12 (31)	27 (22)
2 – 5 years	32 (39)	7 (17)	39 (31)
More than 5 years	7 (8)	0 (0)	7 (6)
Desired difference in children’s ages			
Less than 12 months	1 (1)	0 (0)	1 (1)
12 – 17 months	7 (8)	3 (7)	10 (8)
18 – 23 months	8 (10)	7 (17)	15 (12)
2 – 3 years	41 (49)	24 (57)	65 (52)
3 – 5 years	23 (28)	7 (17)	30 (24)
More than 5 years	3 (4)	1 (2)	4 (3)

* Significant difference between groups, Chi-square, p = 0.020.

**Table 4:** Desired learning environment for IPI information.

	Clinic (n = 83)	NICU (n = 42)	All Participants (n = 125)
Trusted sources for IPI information			
Obstetrician (OB)	53 (66)	38 (90)	91 (75)
Family doctor	54 (68)	30 (71)	84 (69)
Pediatrician	46 (58)	26 (62)	72 (59)
Nurse	32 (40)	24 (57)	56 (46)
Hospital	26 (33)	24 (57)	50 (41)
Family	30 (38)	7 (17)	37 (30)
Midwife	11 (14)	15 (36)	26 (21)
Friends	15 (19)	5 (12)	20 (16)
Doula	3 (4)	10 (24)	13 (11)
Internet	5 (6)	7 (17)	12 (10)
Books/magazines	4 (5)	7 (17)	11 (9)
Television	0 (0)	0 (0)	0 (0)
Other healthcare worker	3 (4)	3 (7)	6 (5)
Other	3 (4)	3 (7)	6 (5)
Best time for IPI information delivery			
Before any pregnancy	32 (39)	22 (52)	54 (44)
During pregnancy	26 (32)	19 (45)	45 (36)
At the time of my baby’s birth (in hospital)	18 (22)	14 (33)	32 (36)
Shortly after my baby’s birth	42 (51)	24 (57)	66 (53)
Other	8 (10)	0 (0)	8 (6)
Best way to deliver IPI information			
Face-to-face discussion	55 (68)	36 (90)	91 (75)
Brochure or handout	35 (43)	22 (55)	57 (47)
Brief video	11 (14)	7 (18)	18 (15)
Email	10 (12)	4 (10)	14 (12)
Social media (e.g., Facebook)	5 (6)	5 (13)	10 (8)
Text message	7 (9)	1 (3)	8 (7)
